# Probiotic characterization and spore production optimization of *Lysinibacillus* sp. MK212927

**DOI:** 10.1007/s00253-026-13755-8

**Published:** 2026-03-08

**Authors:** Sayed E. El-Sayed, Albeir A. Messiha

**Affiliations:** 1 Department of Microbiology and Immunology, Faculty of Pharmacy, Ahram Canadian University, POB: 12451 , Sixth of October City, Giza, Egypt; 2 Department of Inpatient Pharmacy, Virginia Hospital Center, Arlington, VA 22205 USA

**Keywords:** *Lysinibacillus*, Probiotics, Enteropathogens, Response surface methodology, Central composite design, Optimization

## Abstract

**Abstract:**

Members of the genus *Lysinibacillus* are increasingly explored as probiotic candidates, yet thorough screening and safety assessment remain essential due to the toxin-producing potential of related species. In this study, a soil-derived isolate, *Lysinibacillus* sp. MK212927 demonstrated strong antagonistic activity against multiple human enteropathogens and was selected for comprehensive characterization. The strain exhibited high resilience under physiologically relevant stress conditions, including low pH, simulated gastric and intestinal fluids, bile salts, and thermal exposure. It also displayed desirable probiotic attributes such as antioxidant capacity and bile salt hydrolase activity. Safety evaluation revealed the absence of hemolytic activity, minimal cytotoxicity toward Caco-2 cells, and susceptibility to vancomycin, levofloxacin, sulfamethoxazole, and doxycycline, with intermediate susceptibility to azithromycin and amoxicillin, suggesting a lack of plasmids or mobile genetic elements. To enhance its industrial applicability, response surface methodology (RSM) was applied to optimize biomass and spore production. Optimal conditions (pH 6.1, 33.5 °C, 200 rpm, and 0.21 vvm aeration) resulted in a 3.1-fold increase in biomass and a 5.4-fold increase in spore yield. *In vivo* assessment further showed that administration of *Lysinibacillus* sp. MK212927 improved body weight gain in rats, supporting its functional benefits as a feed supplement. Overall, the comprehensive phenotypic and safety evaluations highlight *Lysinibacillus* sp. MK212927 as a robust probiotic candidate with significant potential for controlling enteropathogens and for use in animal and human nutrition, warranting further preclinical and functional development.

**Key points:**

• *Lysinibacillus sp. MK212927 exhibits strong inhibitory activity against human enteropathogens.*

• *The strain tolerates low pH, bile salts, gastric and intestinal fluids, and heat, while displaying antioxidant and bile salt hydrolase activity.*

• *Optimization of biomass and spore production, along with improved body weight in rats, supports its potential as a feed supplement.*

**Supplementary Information:**

The online version contains supplementary material available at 10.1007/s00253-026-13755-8.

## Introduction

According to the definitions provided by the World Health Organization (WHO) and the Food and Agriculture Organization (FAO) of the United Nations, probiotics are live microorganisms that confer health benefits to the host when consumed sufficiently (Hill et al. 2014). Due to their recognized health-promoting effects, probiotics have recently garnered substantial attention, extending beyond the scientific community to encompass growing interest from the food, pharmaceutical, and healthcare sectors. The global probiotic market has expanded beyond scientific and clinical interest to encompass the general consumer sector, including functional foods and beverages fortified with probiotics, dietary supplements, and probiotic-based formulations for animal feed products. Recent market analyses forecast a consistent increase in the global probiotic sector, with an anticipated Compound Annual Growth Rate (CAGR) of approximately 14% between 2023 and 2030 (Lima et al. 2025). Traditionally, lactic acid bacteria (LAB), including genera such as *Lactococcus*, *Bifidobacterium*, and *Lactobacillus*, have been regarded as safe and widely utilized in probiotic formulations (Doron and Snydman 2015). Despite the extensive global use of functional LAB in fermented food products, a significant demand in the biofunctional product industry for new and diversified probiotic formulations is still present. Consequently, considerable research has been directed toward identifying and characterizing novel strains with distinct functional attributes (de Melo Pereira et al. 2018). In recent years, emerging microbial groups, including specific yeasts, additional LAB species, and *Bacillus* strains, have been increasingly explored as promising probiotic candidates (Lee et al. 2022). *Bacillus* species have long been recognized for their industrial importance. They have been extensively employed in large-scale enzyme production and are also widely used as probiotics and direct-fed microbial additives to improve animal health and nutrition. The probiotic potential of these strains is largely attributed to their capacity for endospore formation, which imparts exceptional resilience under harsh environmental conditions (e.g., bile salt exposure and acidic pH) within the gastrointestinal tract (GIT) of humans and animals (Brutscher et al. 2022). Several *Bacillus* strains, such as *B. clausii*,* B. coagulans*, and *B. subtilis*, are widely employed in the pharmaceutical and food industries owing to their spore-forming capacity and established safety profiles (Colom et al. 2021). As is well known, probiotics are scientific terms referring to microorganisms that can contribute to gut microbial balance and exhibit beneficial effects on the health of humans and animals (Soccol et al. 2010). In general, probiotics can pass through the gut tract with high stress tolerance and then show multiple probiotic efforts by rapidly adhering to the intestinal mucosa (Plessas et al. 2017). Recently, *Lactobacillus*, *Bifidobacterium*, and *Bacillus* strains have been widely studied for their ideal traits, including safety assessment, microbial antagonism, and stress tolerance (Soares et al. 2019). In contrast, recent studies indicate that *Lysinibacillus* strain displaying high tolerance against ambient stressors can modulate gut microbial diversity and increase body weight gain in mice (Zeng et al. 2023). *Lysinibacillus* sp. is a spore-forming gram-positive bacterium, and it can survive high temperature, transit gastric and bile juices in the gut, increase the shelf life of feed, and additionally sustain low temperature during storage. *Lysinibacillus* sp. have antimicrobial properties, which makes them more appropriate for aquaculture (Mani et al. 2021). Probiotics primarily exert their beneficial effects within the gastrointestinal tract by creating an environment unfavorable for pathogenic colonization, while simultaneously promoting the growth of beneficial commensal microorganisms. This occurs through the secretion of organic acids, which lower intestinal pH and stimulate the synthesis of antimicrobial metabolites that inhibit pathogenic proliferation. Furthermore, probiotics contribute to maintaining microbial homeostasis by enhancing the population of beneficial bacteria and strengthening host immune defenses. Such activities collectively help protect against intestinal disorders arising from pathogenic infections or physiological stress (Alagawany et al. 2023).

## Materials and methods

### Materials

A previously characterized soil bacterium, *Lysinibacillus *sp*.*, was obtained from earlier isolation and identification work (El-Sayed et al. 2020b). The strain has been deposited in the Culture Collection of Ain Shams University (CCASU) under the accession number CCASU-MK212927 (available at: http://ccinfo.wdcm.org/collection/by_id/1186). The corresponding nucleotide sequence has also been registered in the NCBI GenBank database under the accession number MK212927. The strain colonies were preserved at − 80 °C in 20% sterile glycerol until further use. For activation, the culture was streaked onto commercial de Man, Rogosa, and Sharpe (MRS) agar (HiMedia, Mumbai, India) and incubated at 37 °C.

### Methods

#### Antipathogenic activity by agar well diffusion assay

Antipathogenic activity was evaluated using an agar well diffusion assay adapted from Zhao et al. (2024). Eight human enteropathogenic bacterial strains (*Staphylococcus aureus* ATCC 29213, *Escherichia coli* O157:H7, *Bacillus cereus* ATCC 9634, *Bacillus subtilis* ATCC 6633, *Salmonella enteritidis* ATCC 13076, *Salmonella typhimurium* ATCC 14028, *Campylobacter jejuni* ATCC 29428, and *Clostridium perfringens* ATCC 13124) were cultured under organism-specific optimal conditions and standardized prior to testing. Standardized bacterial suspensions were inoculated onto agar plates, and wells were aseptically prepared and loaded with the actively growing culture of *Lysinibacillus* isolate MK212927. Plates were incubated under appropriate conditions for each pathogen, after which the inhibition zones were measured to assess antimicrobial activity. *Lactobacillus acidophilus* ATCC 43121 was included as a reference probiotic control. Detailed procedures for culture preparation, inoculum standardization, agar well preparation, and zone measurement are provided in the Supplementary Methods (S1).

#### Sensitivity of the bacterium to antibiotics

The antibiotic susceptibility profile of *Lysinibacillus* sp. MK212927 was determined using the standard disc diffusion method on agar plates following Clinical and Laboratory Standards Institute (CLSI) guidelines. The strain was tested against a panel of clinically relevant antibiotics representing different classes. Inhibition zone diameters were measured after incubation, and susceptibility was interpreted according to CLSI criteria. Detailed procedures for inoculum preparation, antibiotic disc specifications, incubation conditions, and zone diameter measurement are provided in Supplementary Methods (S2).

#### Characterization of the antimicrobial activity of *Lysinibacillus* sp.MK212927

To gain preliminary insight into the nature of the antimicrobial activity exhibited by *Lysinibacillus* sp.MK212927, a set of complementary assays was conducted to evaluate whether the observed inhibition was pH-dependent, proteinaceous, or attributable to heat-stable secondary metabolites, as commonly applied in probiotic and antimicrobial characterization studies. Cell-free supernatants (CFS) were obtained by centrifugation of 24-h cultures at 8000 × *g* for 10 min, followed by filtration through 0.22-μm membrane filters to remove residual bacterial cells, to be used in the following assays (pH neutralization, protease sensitivity, and heat stability assay).

#### pH neutralization assay

To assess the contribution of organic acids, aliquots of the CFS were adjusted to pH 7.0 using sterile 1 M NaOH, while parallel aliquots were retained at their native pH. Both neutralized and non-neutralized samples were evaluated for antimicrobial activity using the agar well diffusion assay against human enteropathogenic bacterial strains, *S. aureus* ATCC 29213, *E. coli* O157:H7, *B. cereus* ATCC 9634, *B. subtilis* ATCC 6633, *S. enteritidis* ATCC 13076, *S. typhimurium* ATCC 14028, *Campylobacter jejuni* ATCC 29428, and *Clostridium perfringens* ATCC 13124. Plates were incubated for 24 h under conditions appropriate for each pathogen. A reduction or loss of inhibitory activity following neutralization was interpreted as indicative of acid-mediated antimicrobial effects (Arena et al. 2018; Zommiti et al. 2020).

#### Protease sensitivity assay

To examine the involvement of proteinaceous compounds, CFS aliquots were treated with proteinase K (1–2 mg/mL) and incubated at 37 °C for 1 h. Untreated supernatant and enzyme-buffer controls were included. Residual antimicrobial activity was subsequently assessed by agar well diffusion. A marked decrease in inhibition following protease treatment was considered suggestive of proteinaceous antimicrobial substances, such as bacteriocin-like compounds (Todorov et al. 2020; Vieco-Saiz et al. 2019).

#### Assessment of acid and bile salt tolerance

The tolerance of *Lysinibacillus* sp. MK212927 to acidic and bile salt stress was evaluated by comparing viable cell counts before and after short-term exposure to conditions mimicking the gastrointestinal environment as described earlier (Brutscher et al. 2022). Survival under acidic pH values and varying bile salt concentrations was assessed to determine the strain’s robustness and probiotic suitability.

#### Tolerance to simulated gastrointestinal fluids and thermal stress

The tolerance of *Lysinibacillus* sp. MK212927 to gastrointestinal and thermal stress conditions was evaluated to assess its suitability for probiotic and industrial applications. Resistance to simulated gastric fluid (SGF) and simulated intestinal fluid (SIF) was assessed by comparing viable counts before and after exposure to conditions mimicking the gastrointestinal environment. Thermal tolerance was evaluated by determining cell viability following exposure to elevated temperatures relevant to food and industrial processing. Detailed compositions of SGF and SIF, exposure conditions, temperature treatments, and viability determination procedures are provided in Supplementary Methods (S3).

#### Antioxidant activity (DPPH scavenging assay)

The antioxidant activity of *Lysinibacillus* sp. MK212927 was evaluated using the DPPH radical scavenging assay. Antioxidant capacity was determined spectrophotometrically and expressed as percentage DPPH scavenging activity (AA%). L-ascorbic acid was used as a positive control, while *Lactobacillus acidophilus* ATCC 43121 served as a reference probiotic strain for comparative evaluation. Detailed procedures for reaction setup, incubation conditions, absorbance measurement, and calculation of antioxidant activity are provided in Supplementary Methods (S4).

#### Bile salt hydrolase (BSH) activity assay

A 5 μL aliquot of *Lysinibacillus* culture was inoculated onto two TSA plate sets, one supplemented with taurodeoxycholate hydrate (TDC) (0.5% (w/v)) and another without supplementation, serving as a control. The plates were incubated at 37 °C for 24 h. Bile salt hydrolase (BSH) activity was assessed by observing the formation of an opaque or creamy precipitate on TDC-containing plates after 48 h, indicating bile acid deconjugation. *L. acidophilus* ATCC 43121 served as the probiotic reference strain (Zommiti et al. 2020).

#### Hemolytic activity

*Lysinibacillus* was assessed for hemolytic activity on Columbia agar plates enriched with sheep blood (5% v/v). Cultures of bacteria were streaked over the blood agar plates and kept aerobically at 37 °C for 24–48 h of incubation. The hemolytic pattern was determined by observing the presence and nature of the halo surrounding the colonies. Strains displaying halos were classified as β-hemolytic (total red blood cell lysis), whereas those producing a greenish zone were identified as α-hemolytic (partial hemolysis due to hemoglobin reduction). Colonies exhibiting no visible halo were considered γ-hemolytic (non-hemolytic or weakly hemolytic) (Dabiré et al. 2022).

#### Cell cytotoxicity (CCK‑8 assay)

The cytotoxic potential of *Lysinibacillus* sp. MK212927 was evaluated in Caco-2 cells using the Cell Counting Kit-8 (CCK-8) assay. Cell viability following exposure to cell-free bacterial filtrates was determined spectrophotometrically and expressed as a percentage relative to untreated control cells. *Lactobacillus acidophilus* ATCC 43121 was included as a reference probiotic control. Detailed procedures for sample preparation, assay conditions, controls, and viability calculation are provided in Supplementary Methods (S5).

#### Adhesion capacity assessment

The adhesion capacity of *Lysinibacillus* sp. MK212927 to intestinal epithelial surfaces was evaluated using an *in vitro* adhesion assay with human Caco-2 cells. Adhesion was assessed by quantifying viable bacteria attached to differentiated Caco-2 monolayers following co-incubation. Adhesion capacity was expressed as the percentage of bacteria adhered relative to the initial inoculum. Detailed procedures for cell culture conditions, bacterial preparation, adhesion assay steps, and enumeration are provided in Supplementary Methods (S6).

### Submerged fermentation for spore production

#### Inoculum preparation

A loopful of the activated culture was transferred into a 250-mL Erlenmeyer flask containing 50 mL of MRS broth (HiMedia, Mumbai, India) and incubated at 37 °C with shaking at 200 rpm for 12 h. Cells were collected by centrifugation at 2500 × *g* for 10 min at 4 °C, washed twice with 0.85% NaCl, and resuspended in the same solution to an OD₆₀₀ of 1.0 for use as the inoculum (Su et al. 2019).

### Fermentation medium and conditions

The fermentation medium consisted of triammonium citrate (5 g/L), glucose (10 g/L), peptone (10 g/L), yeast extract (10 g/L), sodium acetate (5 g/L), phosphate buffer, and trace elements (Ca^2^⁺, Mn^2^⁺, Mg^2^⁺, Fe^2^⁺, Co^2^⁺) supplied from stock solutions. Glucose and the phosphate buffer were sterilized separately before addition to the medium. The pH was adjusted according to the experimental design for each trial (Sen and Babu 2005).

### Optimization of biomass and spore production by response surface methodology

#### Experimental design

Response surface methodology (RSM) was applied using a face-centered central composite design (CCD) in Design Expert® v11.0 (Stat-Ease Inc., Minneapolis, MN, USA). Thirty experiments, including six center points for error estimation, were conducted. The four independent factors were pH (A), temperature (°C, B), agitation rate (rpm, C), and aeration rate (vvm, D). Biomass yield (g L⁻^1^) and spore yield (g⁻^1^) served as the response variables. Each factor was tested at three levels, as summarized in Table 1.

**Table 1:** Experimental ranges of variables used in CCD optimization

Independent variables	Level of variables		
	**−1**	**0**	** + 1**
A: pH	5	6.5	8
B: Temperature °C	30	37	44
C: Agitation rate (rpm)	100	150	200
D: Aeration rate (vvm)	0.2	0.5	0.8

#### Biomass determination

Biomass concentration was determined spectrophotometrically at 600 nm using a UV–Vis spectrophotometer (Shimadzu 1700, Japan). Broth samples were collected every 4 h and diluted appropriately to maintain absorbance values below 0.6 (Khardziani et al. 2017). The detailed assessment of biomass and spore production is fully explained in Supplementary Methods (S7).

#### *In vivo* maintenance and experimental treatments

All experimental procedures complied with institutional and international guidelines for the care and use of laboratory animals and the ARRIVE guidelines (https://arriveguidelines.org; accessed 20 March 2024). Twenty-five adult male Wistar rats (220–240 g) were obtained from the Animal House, Faculty of Pharmacy, Ahram Canadian University (Giza, Egypt), and acclimatized for 15 days before experimentation. The protocol was approved by the Faculty’s Research Ethics Committee (Approval No. ACUC-FP-ACU-REC#1524). The rats were assigned to five groups on a random basis (*n* = 5 per group) as follows:

- Group (I): Control, fed with a standard compound diet.
- Group (II): Treatment, fed with antibiotic 100 g/t oxytetracycline.
- Group (III): Treatment, fed with *Lysinibacillus* (1.0 × 10^6^ CFU/mL).
- Group (IV): Treatment, fed with *E. coli* O157:H7 (1.0 × 10^6^ CFU/mL).
- Group (V): Control, fed with *L. acidophilus* ATCC 43121 (1.0 × 10^6^ CFU/mL).

Rats were maintained under previously reported housing conditions, and the composition of the basal diet followed that described in the earlier study (Xu et al. 2023). Body weight was recorded before feeding and at 7-day intervals, and animals were regularly monitored for adverse effects such as diarrhea.

### Statistical analysis

Statistical analysis was performed using one-way ANOVA followed by Tukey’s multiple comparison test (GraphPad Software Inc., San Diego, CA, USA) to calculate *p*-values and standard deviations. Results were expressed as mean ± standard deviation. Response surface modeling, data analysis, and diagnostic plotting were conducted using Design-Expert® v11.0. The experimental model was statistically validated by ANOVA to assess the significance of individual parameters.

## Results

### Antipathogenic activity by agar well diffusion assay

*Lysinibacillus* isolates demonstrated strong inhibitory activity against several human enteropathogens (Table 2). Notably, *Lysinibacillus* effectively suppressed the growth of major foodborne pathogens, including *S. aureus*, *B. cereus*, and the virulent serotype *E. coli* O157:H7, all associated with diarrheal diseases. In addition, the strain exhibited inhibitory effects against both Gram-positive and Gram-negative bacteria, such as *C. perfringens*, *S. enteritidis*, and *S. typhimurium*.

**Table 2:** Inhibition zones of *Lysinibacillus* sp. MK212927 and *L. acidophilus* ATCC 43121 against tested enteropathogens

Strain	Diameter of inhibition zone (mm)							
	***S. aureus* ATCC 29213**	***E. coli* O157:H7**	***B. cereus* ATCC 9634**	***B. subtilis *ATCC 6633**	***Salmonella enteritidis* ATCC 13076**	***Salmonella typhimurium* ATCC 14028**	***Clostridium perfringens* ATCC 13124**	***Campylobacter jejuni* ATCC29428**
***Lactobacillus acidophilus* ATCC 43121**	14.5 ± 0.11	11.5 ± 0.45	14.2 ± 0.5	12.6 ± 0.7	11.6 ± 0.7	13.8 ± 0.43	15.5 ± 0.65	11.4 ± 0.71
***Lysinibacillus* sp. MK212927**	16.4 ± 0.44	15.1 ± 0.56	16.2 ± 0.33	14.7 ± 0.63	13.6 ± 0.71	15.8 ± 0.89	17.4 ± 0.91	12.5 ± 0.24

### Sensitivity of the bacterium to antibiotics

The antibiotic susceptibility of the bacterial strain in the present study was determined. It indicates that this *Lysinibacillus* strain is susceptible to vancomycin (30 µg), levofloxacin (5 µg), sulfamethoxazole (25 µg), streptomycin (25 µg), and doxycycline (30 µg), with intermediate sensitivity to azithromycin (15 µg) and amoxicillin (25 µg). This is considered a promising result concerning the safety of probiotic application because of the consequent problem, probably due to the horizontal transfer of antibiotic resistance genes to non-related pathogenic or commensal bacteria within the gut microbiota.

### Characterization of the antimicrobial activity of *Lysinibacillus* sp.MK212927

#### pH neutralization assay

Neutralization of the *Lysinibacillus* sp.MK212927 cell-free supernatant to pH 7.0 did not result in a significant reduction in antimicrobial activity against the tested enteropathogens. Comparable inhibition zone diameters were observed for both native and pH-neutralized supernatants, indicating that the antagonistic effect was not primarily mediated by organic acids and was therefore largely pH-independent.

#### Protease sensitivity assay

Treatment of the cell-free supernatant with proteinase K did not lead to a noticeable decrease in antimicrobial activity. Inhibition zones produced by protease-treated samples remained comparable to those obtained with untreated supernatants, suggesting that the inhibitory compounds were not proteinaceous in nature, and these observed properties are inconsistent with proteinaceous bacteriocins and instead suggest the involvement of heat-stable, non-protein secondary metabolites.

#### Assessment of acid and bile salt tolerance

**Fig. 1:**
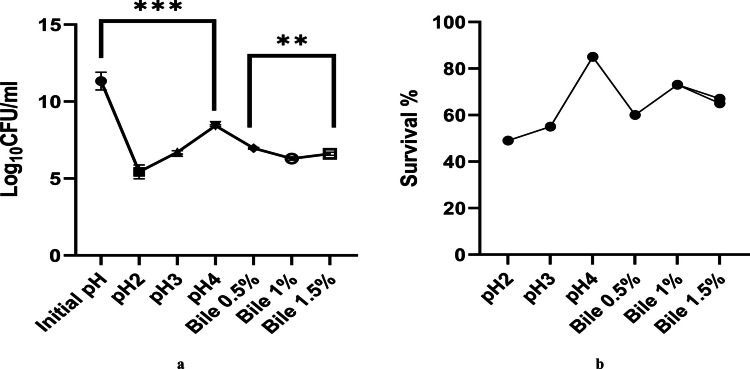
**a** Log_10_ CFU/mL of *Lysinibacillus* sp. MK212927 was measured before and after exposure to acid or ox-bile salt for 2 h. **b** *Lysinibacillus* sp. MK212927 tolerance to acid and bile salt

**Fig. 2:**
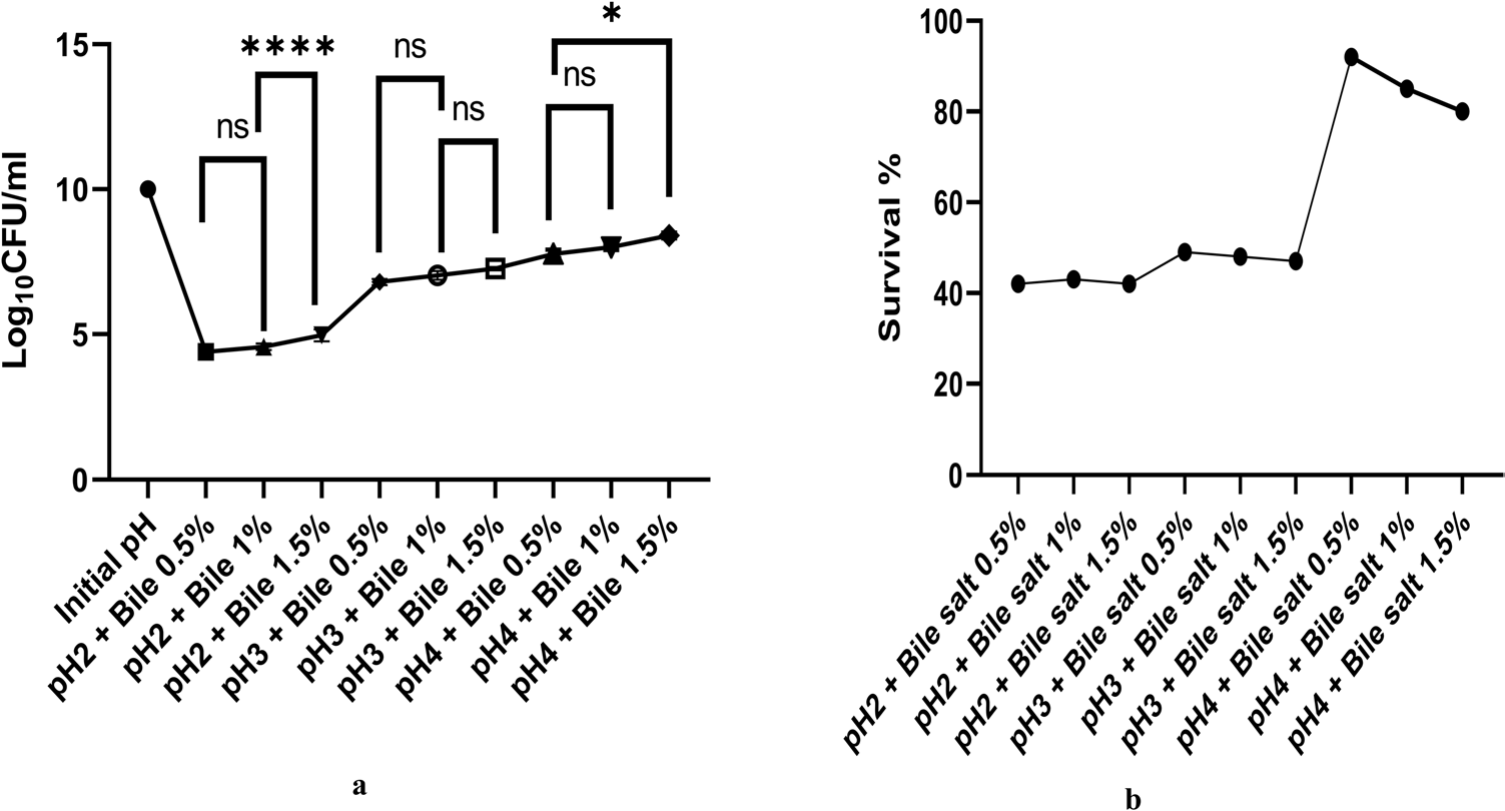
**a** Log_10_ CFU/mL of *Lysinibacillus* sp. MK212927 was measured before and after exposure to acid mixed with ox-bile salts for 2 h. **b** *Lysinibacillus* sp. MK212927 survival % in mixed acid and bile salt conditions

The survival of *Lysinibacillus* under acidic conditions (pH 2, 3, and 4), varying ox-bile salt concentrations (0.5%, 1.0%, and 1.5% w/v), and combined acid–bile stress for 2 h is shown in Fig. 1a, b and Fig. 2a, b. The strain exhibited marked sensitivity at pH 2, with a reduction of 4–5 Log₁₀ CFU/mL. At pH 3 and 4, however, the decline was less than 1 Log₁₀ CFU/mL, indicating strong acid tolerance above pH 3. Exposure to bile salts resulted in 2–3 Log₁₀ CFU/mL reductions across concentrations, reflecting high bile resistance. Notably, at pH 3 with bile salts, viable counts consistently exceeded 6 Log₁₀ CFU/mL, suggesting the strain’s ability to survive and potentially colonize the gastrointestinal tract. Under combined acid–bile stress, the survival trend closely resembled that under acidic conditions alone, highlighting acid as the primary factor influencing *Lysinibacillus* viability.

#### Tolerance to simulated gastrointestinal fluids and thermal stress

**Fig. 3:**
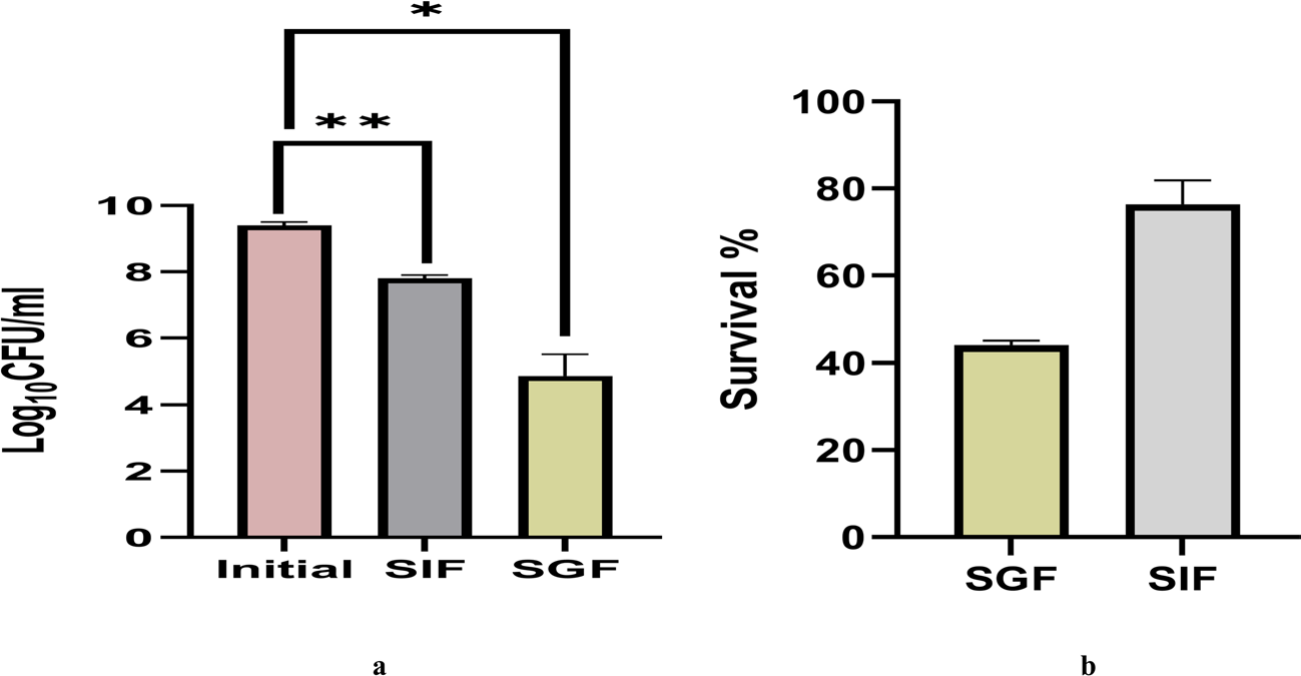
**a** Log_10_ CFU/mL of *Lysinibacillus* sp. MK212927 was measured in SGF and SIF. **b** *Lysinibacillus* sp. MK212927 survival % in SGF and SIF

**Fig. 4:**
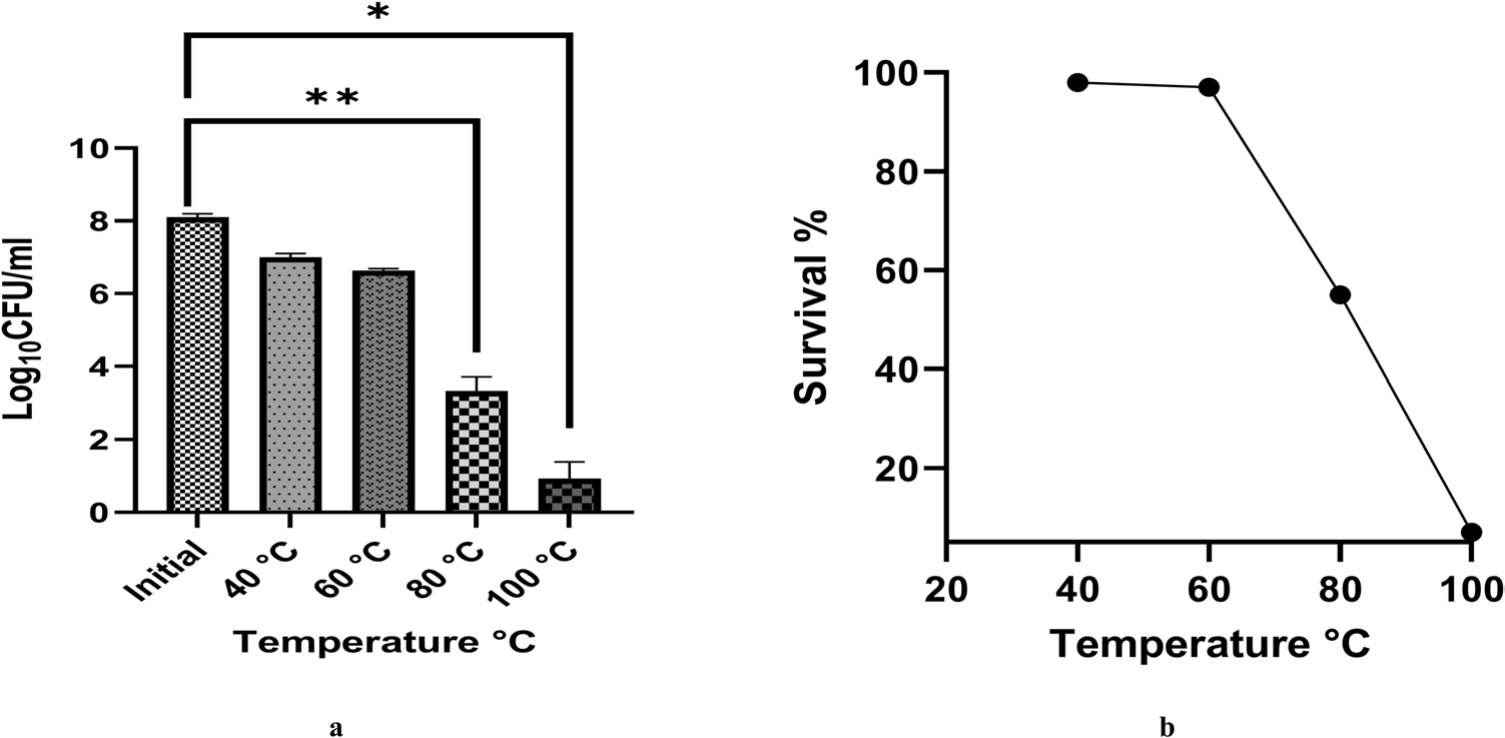
*Lysinibacillus *sp. MK212927 tolerance to elevated temperatures (40 °C–100 °C)

The tolerance of *Lysinibacillus* to simulated gastric fluid (SGF) and simulated intestinal fluid (SIF) was evaluated to mimic gastrointestinal conditions, and the results are shown in Fig. 3a, b. The strain exhibited strong survival in SIF, maintaining counts above 8 Log₁₀ CFU/mL after 2 h. In contrast, exposure to SGF (pH 2, containing porcine pepsin) led to a 4–5 Log₁₀ CFU/mL reduction, indicating moderate acid sensitivity. Despite this decline, survival remained around 50%, demonstrating good overall tolerance to both SGF and SIF. These findings highlight the probiotic potential of *Lysinibacillus* and suggest that encapsulation could further improve its viability within the human gastrointestinal tract. The results of the heat stability assay are illustrated in Fig. 4a, b. As shown, bacterial viability exhibited minimal variation at 40 °C, with only a one-log₁₀ CFU/mL reduction observed. When the temperature was increased to 60 °C, a slight decrease in viable count occurred, with Log₁₀ CFU/mL values declining from 8.1 to 7.9, corresponding to a survivability rate exceeding 90%. In contrast, exposure to 100 °C caused a sharp decline in viable cell counts, reducing them to 1.7 Log₁₀ CFU/mL, indicating significant thermal inactivation at this temperature.

#### Antioxidant activity (DPPH scavenging assay)

**Fig. 5:**
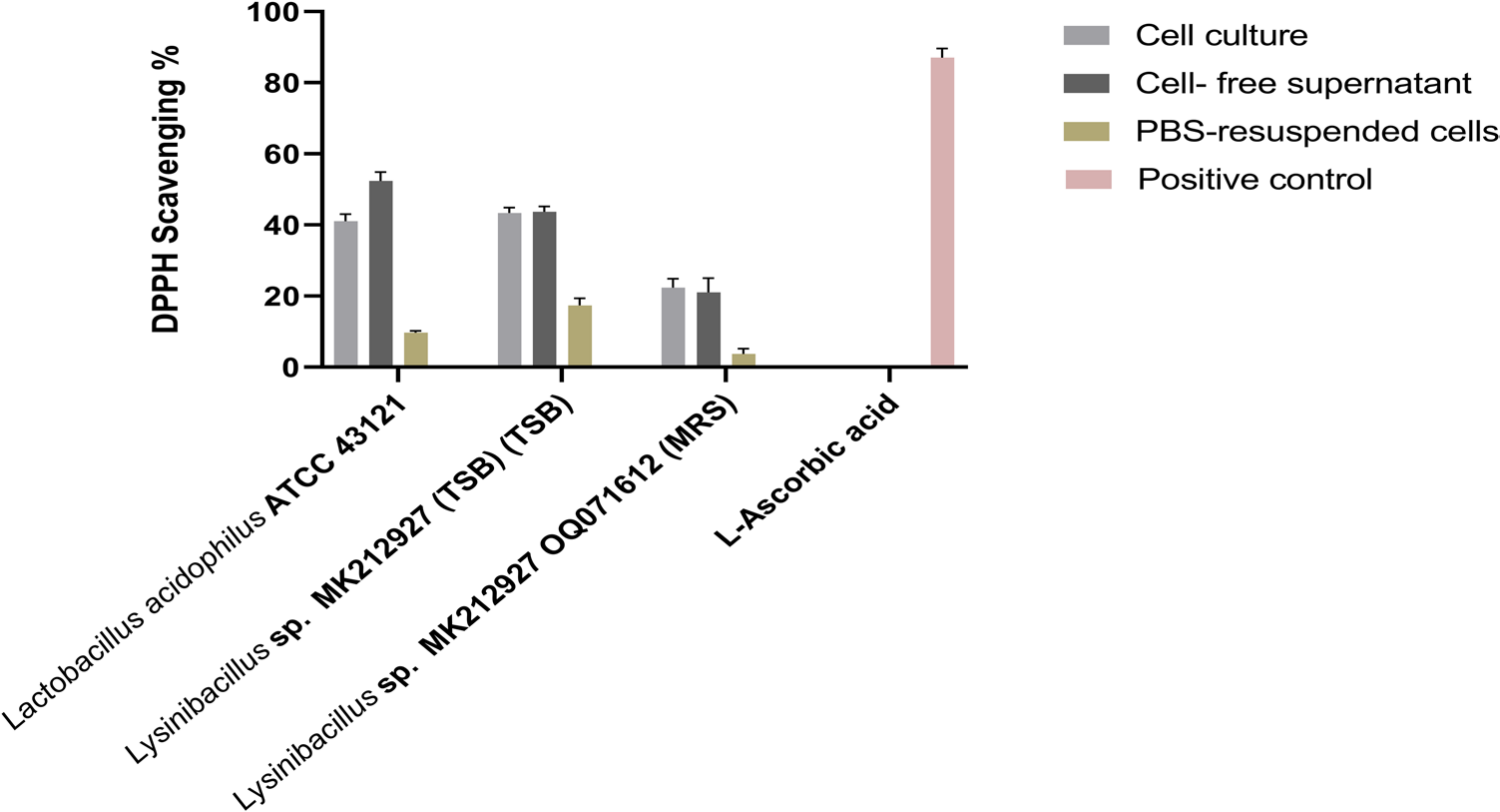
Antioxidant activity of *Lysinibacillus *sp. MK212927 cultured in TSB and MRS broth

**Fig. 6:**
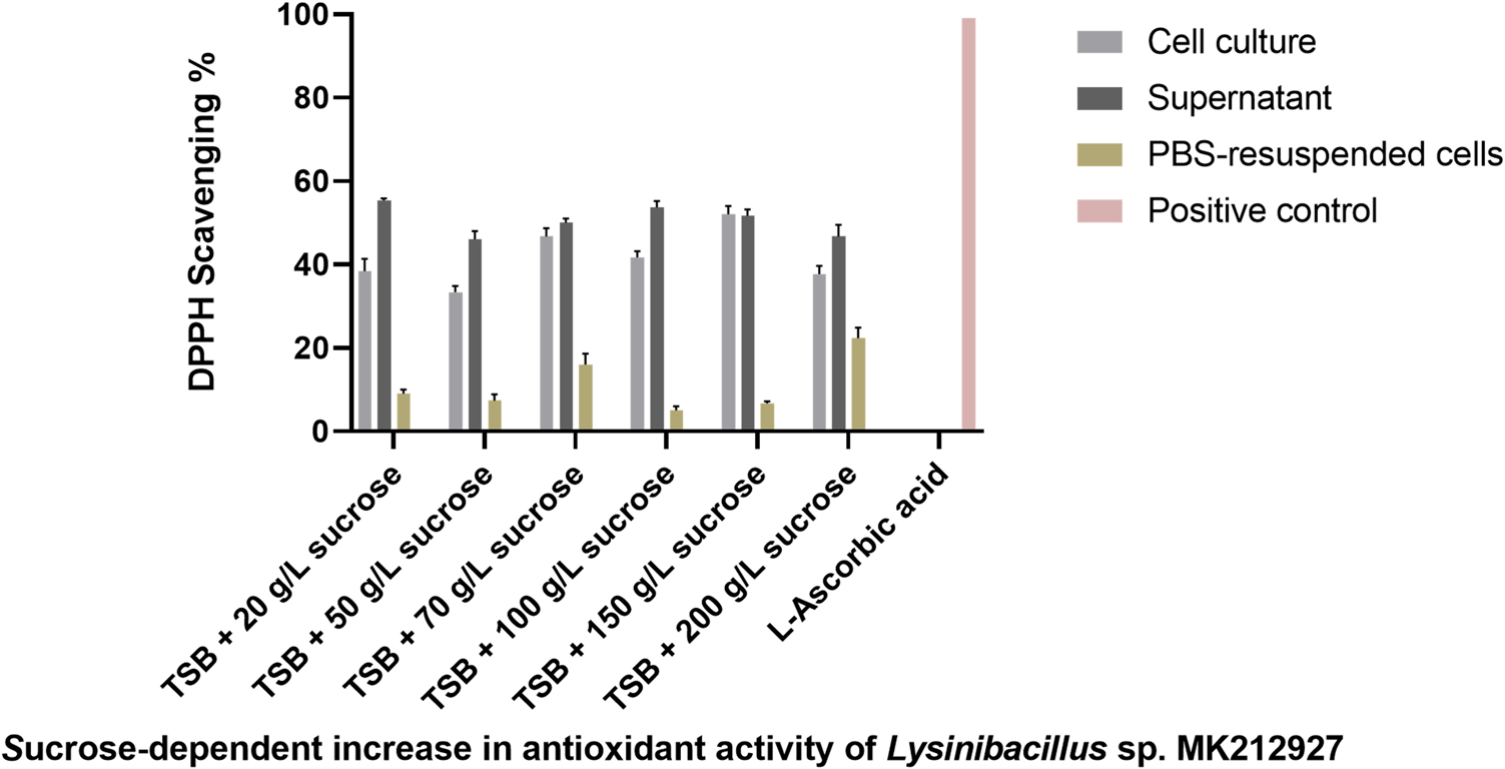
Antioxidant activity of *Lysinibacillus* sp. MK212927 in TSB supplemented with different concentrations of sucrose

The antioxidant activities of the cell culture, supernatant, and PBS-resuspended cells of *Lysinibacillus* are presented in Figs. 5 and 6. As shown in Fig. 6, *Lysinibacillus* grown in TSB exhibited strong antioxidant activity, with approximately 40% DPPH radical scavenging, comparable to *L. acidophilus* ATCC 43121. In contrast, cells cultured in MRS broth showed lower antioxidant activity, with 10–15% DPPH scavenging. PBS-resuspended cells displayed reduced activity relative to the original culture and supernatant, indicating that the antioxidant capacity mainly arises from extracellular metabolites such as exopolysaccharides (EPS) and organic acids. Growth in MRS, a low-pH medium optimized for lactic acid bacteria, appears to promote biofilm formation, a microbial stress adaptation mechanism rather than one favoring secondary metabolite synthesis. Additionally, Fig. 6 demonstrates a sucrose-dependent increase in antioxidant activity, with DPPH scavenging rising from 40 to 60% as the sucrose concentration in TSB increased to 150 g/L, beyond which no further enhancement was observed. These findings suggest that sucrose, rather than monosaccharides, plays a key role in EPS-mediated antioxidant activity.

#### Bile salt hydrolase (BSH) activity assay

*Lysinibacillus* exhibited clear bile salt hydrolase (BSH) activity, evidenced by distinct white precipitates surrounding colonies on TDC-supplemented plates. No such changes were observed on control TSA plates without TDC. The formation of the white halo indicates enzymatic deconjugation of conjugated bile salts into their primary forms, confirming active BSH functionality in the tested strain.

#### Hemolytic activity

The isolated *Lysinibacillus* strain exhibited γ-hemolysis, indicating a non-hemolytic pattern.

#### Cell cytotoxicity (CCK‑8 assay)

The cytotoxic effects of the cell-free filtrates from *Lysinibacillus* and *L. acidophilus* ATCC 43121 are shown in Fig. S1. Both filtrates exhibited minimal cytotoxicity toward Caco-2 cells. At concentrations of 5 µL/mL or 10 mg/mL, a slight inhibition of Caco-2 cell proliferation was observed, likely due to bioactive metabolites and hydrogen peroxide produced by *Lysinibacillus*. The replacement of DMEM with the fermented medium also contributed to this inhibitory effect. Overall, the low cytotoxicity of both strains supports their safety and potential as probiotic candidates, with *Lysinibacillus* demonstrating comparatively superior performance.

#### Adhesion capacity assessment

The adhesion abilities of *Lysinibacillus* and *L. acidophilus* ATCC 43121 to Caco-2 cells are shown in Fig. S2. Adhesion levels were independent of the initial seeding concentration, with optimal adherence observed at 10⁷ CFU/mL. At this concentration, *Lysinibacillus* exhibited an adhesion rate of approximately 70%, markedly higher than that of *L. acidophilus* ATCC 43121 (20%). The strong adhesion of *Lysinibacillus* likely reflects its enhanced biofilm-forming and surface-binding capabilities. Moreover, *Lysinibacillus* consistently maintained adhesion values above 10⁶ CFU/mL across all tested concentrations, meeting the established probiotic efficacy threshold.

### Optimization of biomass and spore production by response surface methodology

The actual values of the tested factors, experimental design, and corresponding results are presented in Table S1. The regression equation, expressed in coded factors, enables the prediction of the response at specific factor levels. In this model, high and low factor levels are coded as + 1 and − 1, respectively. This coded form facilitates comparison of factor coefficients to assess their relative influence on the response.

The quadratic model equations generated from the central composite design (CCD) using Design-Expert® software are presented as follows:

$$Biomass\;yield=\;44.10+2.26^\ast A+5.06\ast B+5.59^\ast C+2.21^\ast D-9.02^\ast A^2-9.87^\ast B^2$$

$$Spore\;yield=\;7.98-1.75^\ast A-3.40^\ast B+0.1628^\ast C-0.3361^\ast D+0.7312^\ast AB+0.0637^\ast AC+0.135^\ast AD+0.0075^\ast BC+0.9137^\ast BD-0.0312^\ast CD$$

The analysis of variance (ANOVA) demonstrated that the developed models for biomass yield (g L⁻^1^) and spore yield (g⁻^1^) were highly significant, with *F*-values of 247.53 and 727.06, respectively (*p* < 0.0001). For biomass yield, the significant model terms included *A* (pH), *B* (temperature), *C* (aeration), *D* (agitation), *A*^2^, and *B*^2^ (*p* < 0.05). For spore yield, the significant terms were A, B, C, D, AB, AD, and BD (*p* < 0.05) as shown in Table 3. The coefficients of variation were 4.63% for biomass yield and 2.49% for spore yield, indicating good experimental precision. The coefficients of determination (*R*^2^) were 0.98 for biomass yield and 0.997 for spore yield, with predicted *R*^2^ values (0.974 and 0.988, respectively) in close agreement with the corresponding adjusted *R*^2^ values (0.98 and 0.996). Adequate precision values of 54.0498 and 106.0863 were obtained for biomass and spore yield models, respectively.

**Table 3:** ANOVA results for the biomass yield (g L⁻^1^) and the spore yield (g⁻^1^)

Source	Sum of squares	df	Mean square		*F*-value	*p*-value		
**Biomass yield (g L⁻**^**1**^**) model**	3415.67	6	569.28		247.53	< 0.0001		Significant
A-pH	91.58	1	91.58		39.82	< 0.0001		
B-Temperature °C	461.07	1	461.07		200.48	< 0.0001		
C-Agitation rate (rpm)	562.24	1	562.24		244.47	< 0.0001		
D-Aeration rate (vvm)	88	1	88		38.26	< 0.0001		
A^2^	280.2	1	280.2		121.83	< 0.0001		
B^2^	335.5	1	335.5		145.88	< 0.0001		
**Residual**	52.9	23	2.3					
Lack of fit	52.26	18	2.9		22.92	0.0013		
Pure error	0.6333	5	0.1267					
**Cor total**	3468.56	29						
**Spore yield (g⁻**^**1**^**) Model**	287.42	10	28.74	727.06			< 0.0001	Significant
A-pH	54.88	1	54.88	1388.27			< 0.0001	
B-Temperature °C	207.74	1	207.74	5255.06			< 0.0001	
C-Agitation rate (rpm)	0.4769	1	0.4769	12.06			0.0025	
D-Aeration rate (vvm)	2.03	1	2.03	51.44			< 0.0001	
AB	8.56	1	8.56	216.43			< 0.0001	
AC	0.065	1	0.065	1.64			0.2151	
AD	0.2916	1	0.2916	7.38			0.0137	
BC	0.0009	1	0.0009	0.0228			0.8817	
BD	13.36	1	13.36	337.93			< 0.0001	
CD	0.0156	1	0.0156	0.3953			0.537	
**Residual**	0.7511	19	0.0395					
Lack of fit	0.7511	14	0.0536					
Pure error	0	5	0					
**Cor total**	288.17	29						

**Fig. 7:**
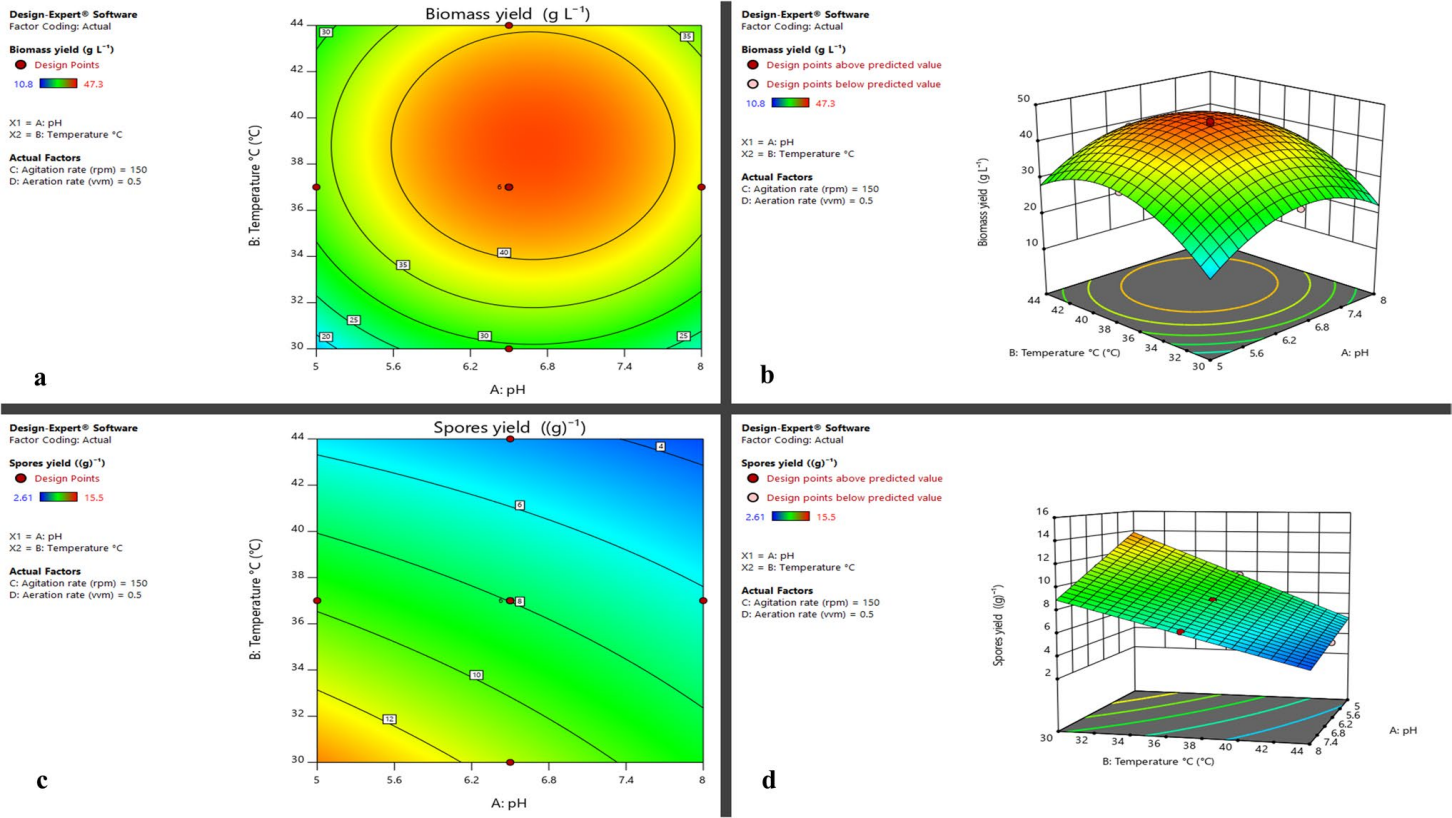
**a** Contour plot, **b** three-dimensional response surface plot representing the effect of the four significant parameters on biomass yield (g L⁻^1^), **c** contour plot, and **d** three-dimensional response surface plot representing the effect of the four significant parameters on spores’ yield (g)⁻^1^

Three-dimensional response surface and contour plots (Fig. 7) indicated that the optimal conditions for maximizing both biomass and spore production were pH 6.1, temperature 33.5 °C, agitation 200 rpm, and aeration 0.21 vvm. Model diagnostic plots (for both biomass and spore yield), including normal probability plots, Box–Cox plots, predicted versus actual plots, and residuals versus run plots, are presented in the Supplementary Figures (Figs. S3–S6) respectively.

Verification experiments conducted under optimized conditions resulted in a 3.1-fold increase in biomass production and a 5.4-fold increase in spore yield. Under these conditions, the biomass concentration reached 40.736 g L⁻^1^, while the spore yield reached 11.1391 × 10^9^ spore g⁻^1^.

### *In vivo* maintenance and experimental treatments

All experimental groups exhibited comparable initial body weights (~ 220 g), confirming uniformity before intervention Table S2. Progressive differences in body weight gain were observed during the three-week feeding period among the control, antibiotic-treated, probiotic-supplemented, and *E. coli* O157:H7–infected groups.

After the first week of feeding, the control group demonstrated a normal increase in body weight (236.0 ± 7.42 g). In contrast, the antibiotic-treated group exhibited a slightly lower gain (230.0 ± 13.42 g), suggesting a transient alteration of the gut microbiota. In contrast, rats supplemented with *Lysinibacillus* (242.6 ± 9.73 g) or *L. acidophilus* ATCC 43121 (230.2 ± 10.73 g) showed comparable or improved weight gain relative to controls, indicating a positive impact on early feed utilization and metabolism. Meanwhile, *E. coli* O157:H7–challenged rats exhibited a notable decline in body weight (213.0 ± 7.58 g; *p* < 0.05), reflecting infection-induced stress and intestinal disturbance.

By the second week, the divergence between treatment groups became statistically significant. The control and antibiotic groups maintained modest weight increases (239.0 ± 11.51 g and 236.0 ± 23.02 g, respectively). However, both probiotic-supplemented groups demonstrated marked improvements in growth performance, with *Lysinibacillus* reaching 269.0 ± 11.32 g and *L. acidophilus* attaining 259.0 ± 16.34 g (*p* < 0.05 compared to the control). In contrast, the *E. coli*-infected group exhibited a continued decline in weight (200.6 ± 19.17 g; *p* < 0.01), indicating a progressive infection and impaired nutrient absorption. In the third week, differences among treatments were more pronounced. The control and antibiotic groups achieved final body weights of 243.2 ± 12.49 g and 239.8 ± 37.75 g, respectively, with *p* > 0.05 between them. Conversely, *Lysinibacillus*-treated rats exhibited a substantial weight increase to 355.3 ± 21.72 g (*p* < 0.01 vs. control), followed by *L. acidophilus* (334.6 ± 19.54 g; *p* < 0.01). The *E. coli*-infected rats continued to lose weight, reaching 189.9 ± 34.85 g by week 3, consistent with infection-induced anorexia and systemic inflammation. Overall, *Lysinibacillus* supplementation resulted in the highest cumulative weight gain among all groups, indicating superior growth-promoting efficacy compared with *L. acidophilus*, antibiotic, and control treatments.

## Discussion

Numerous members of the *Bacillaceae* family, such as *B. subtilis*, *B. licheniformis*, and *B. coagulans*, have long been employed as probiotic agents in nutritional formulations for human and animal use (Chaucheyras-Durand and Durand 2009). Nonetheless, a thorough assessment of their safety is essential, since certain *Bacillaceae* species, such as *B. anthracis* and *B. cereus*, are known to be pathogenic and can induce infections in both humans and animals (Little and Ivins 1999). This study demonstrates the probiotic potential of *Lysinibacillus*, a soil-derived isolate. The strain showed strong inhibitory effects against human enteropathogens and possessed several attributes consistent with probiotic functionality. Although *Lactobacillus acidophilus* ATCC 43121 served as a well-established probiotic reference strain, the antimicrobial activity exhibited by *Lysinibacillus* sp. MK212927 was comparable against several tested enteropathogens. Importantly, this inhibition reflects the action of extracellular antimicrobial metabolites rather than direct competitive exclusion by live probiotic cells. Therefore, the observed inhibition zones are considered biologically meaningful, particularly in the context of spore-forming probiotics, which may exert functional benefits through metabolite production and enhanced environmental stability. Synergistic effects mediated by extracellular metabolites have been reported for *Bacillus spizizenii* and *Bacillus subtilis*, where secreted compounds promoted the growth of companion strains during co-culture, highlighting the importance of metabolite-driven compatibility in multi-strain probiotic formulations (Maniya et al. 2024). *Lysinibacillus* displayed high stress tolerance, beneficial host-associated properties, and a favorable preclinical safety profile. Its stability under acidic and bile conditions, as well as its heat resistance, is largely linked to its biofilm-forming capability, which provides protection against environmental stress and enhances mucosal adhesion features that support its potential use in biomedical and nutraceutical applications. The significant adhesion of *Lysinibacillus* suggests a high capacity for interaction with intestinal epithelial surfaces, a feature commonly associated with effective probiotic strains (Wang et al. 2020). Moreover, *Lysinibacillus* demonstrated consistently strong performance across all tested stress conditions, underscoring its robustness and suitability as a probiotic candidate. The bile salt hydrolase (BSH) activity exhibited by *Lysinibacillus* underlies its potential cholesterol-lowering capability. BSH enzymes cleave the amid bonds of conjugated bile acids, generating unconjugated forms with lower solubility that readily precipitate in acidic environments. Consequently, these free bile acids are more readily excreted in feces. This enhanced excretion stimulates compensatory de novo bile acid synthesis from cholesterol, thereby increasing cholesterol utilization and reducing its circulating levels. Furthermore, bile salt deconjugation decreases the solubility and emulsification efficiency of cholesterol, limiting its intestinal absorption. Collectively, these mechanisms contribute to the hypocholesterolemic effect associated with *Lysinibacillus* and reinforce its potential as a functional probiotic with cardiometabolic health benefits (Modasiya et al. 2024). Antioxidants have attracted considerable scientific and industrial interest due to their diverse biological roles, including anti-aging, anti-inflammatory, and cytoprotective effects, and are widely applied in food technology to enhance product stability and nutritional value. The antioxidant activity demonstrated by *Lysinibacillus* sp. in the present study highlights its potential applicability in pharmacological, cosmetic, and biomedical contexts where mitigation of oxidative stress is essential (Chauhan et al. 2025). Importantly, the antioxidant assays were conducted using crude preparations, and therefore the observed activity likely reflects a combined contribution of both cell-associated components and extracellularly secreted products, rather than a single defined fraction. The safety profile of *Lysinibacillus* sp. was further evaluated using Caco-2 cell line models to assess cytotoxicity relevant to potential probiotic or therapeutic applications. The results showed that the cell-free filtrate exhibited minimal cytotoxic effects toward Caco-2 cells under standard conditions, indicating acceptable biocompatibility at lower concentrations. At higher concentrations, however, a concentration-dependent reduction in cell proliferation was observed. This effect may be associated with the presence of secreted bioactive metabolites, including antimicrobial compounds and reactive oxygen species such as hydrogen peroxide, which, although potentially inhibitory at elevated levels, have also been reported to exert antimicrobial and anticancer activities. It should be noted that, in the absence of fractionation between cell-associated constituents (e.g., cell wall-bound polymers) and extracellular metabolites (e.g., soluble metabolites or exopolysaccharides), the precise source of the antioxidant and cytotoxic effects cannot be conclusively determined. Similar growth-inhibitory effects on colon cancer cell lines have been reported for several lactic acid bacteria strains and their metabolic products, including *Lactobacillus rhamnosus* GG, *L. casei* M3, and *L. plantarum* YYC-3 (Yue et al. 2020). Collectively, these findings suggest that the observed cytotoxic response may reflect a biologically active interaction between bacterial products and host cells, rather than inherent toxicity, while highlighting the need for future studies employing separate analysis of cellular fractions and extracellular metabolites to clarify the mechanisms involved.

Regarding hemolytic activity, *Lysinibacillus* exhibited no hemolysis, which could initially raise concerns about its suitability for use in food or feed applications. However, previous research has established that only β-hemolytic *Bacillus* strains are contraindicated for probiotic development due to their association with pathogenicity (Luise et al. 2022). The hemolytic pattern observed in *Lysinibacillus* is generally considered benign and is also characteristic of several safe and commercially utilized *Bacillus* strains, including *B. subtilis* ATCC 6051. Furthermore, similar findings have been reported in *Lactobacillus* species used in probiotic formulations, such as kefir-derived isolates, where the presence of the *hlyIII* gene encoding a hemolysin-like protein is not regarded as a major safety concern (Sarikkha et al. 2015). Reports of probiotic-related bacteremia remain exceedingly rare (Yelin et al. 2019), emphasizing the minimal risk of systemic translocation following oral administration (Brutscher et al. 2022). Collectively, these findings, supported by the observed low cytotoxicity in Caco-2 cell models, reinforce the safety of *Lysinibacillus* and its significance as a functional probiotic candidate. Antimicrobial resistance (AMR) mechanisms have undergone significant diversification throughout bacterial evolution. Some of these resistance mechanisms evolved as adaptive responses to natural antimicrobial agents, while others developed to fulfill specific physiological roles. The latter are categorized as intrinsic resistance mechanisms, which differ from acquired resistance in being chromosomally encoded, species-specific, and non-transferable through horizontal gene transfer. Instead, they are maintained through clonal propagation within the bacterial population. Therefore, when a species exhibits inherent insensitivity to an antimicrobial compound termed intrinsic resistance, it is regarded as a stable, natural trait rather than a potential safety concern. In contrast, acquired resistance occurs when a typically susceptible bacterial strain gains resistance to an antimicrobial agent, usually through the horizontal transfer of resistance genes carried on plasmids, transposons, or other mobile genetic elements. The emergence of such resistance requires comprehensive molecular and phenotypic characterization to assess its potential clinical and safety implications (on Additives et al. 2018).

For *Lysinibacillus*, susceptibility to the tested antimicrobial agents was observed, indicating no major safety concerns regarding its potential use as a probiotic or in biotechnological applications. Moreover, *Lysinibacillus* is predicted to possess the genetic capacity to synthesize a wide array of bioactive secondary metabolites, particularly antimicrobial compounds with promising therapeutic and industrial value. This metabolic versatility reinforces its biotechnological potential and positions it as a valuable source of naturally derived antimicrobial agents. Previous studies have also documented its antifungal activity against *Candida albicans* ATCC 10231 and *Aspergillus niger* (El-Sayed et al. 2020b). RSM is a widely applied statistical and mathematical tool used to optimize bioprocess conditions and improve microbial biomass or metabolite production. For example, Chen et al. (2010) employed RSM to enhance the viable cell yield of *Bacillus subtilis* WHK from (2.7 ± 0.75) × 10⁹ to (1.52 ± 0.06) × 10^1^⁰ CFU/mL, reflecting a marked improvement in productivity (Chen et al. 2010). Likewise, Sun et al*.* utilized RSM to optimize fermentation conditions for *Pichia pastoris* GS115, resulting in a 2.1-fold increase in xylanase production (Sun et al. 2020). A response surface design was employed in this study to optimize biomass yield and spore production by *Lysinibacillus*. Conventional fermentation optimization approaches, particularly those focused on physical and nutritional factors, are often time-consuming, labor-intensive, and costly, and they fail to consider interactions among multiple variables (Li et al. 2009). Statistical optimization techniques such as RSM offer significant advantages by enabling rapid, reliable analysis of nutrient effects at varying concentrations while reducing the number of required experiments, thereby conserving time, reagents, and resources (Vaidya et al. 2003). RSM facilitates simultaneous evaluation of multiple environmental and nutritional parameters, improving understanding of variable interactions and their influence on metabolite production (Singh et al. 2013). It has been extensively applied in optimizing fermentation conditions and medium compositions to maximize metabolite yields in soil-derived bacteria (Mechri et al. 2017). For instance, Ahsan et al. (2017) used a central composite RSM design to enhance the antifungal activity of *Streptomyces diastatochromogenes* KX852460 through nutrient optimization in submerged fermentation (Ahsan et al. 2017). Similarly, Li et al. (2020) applied RSM to optimize co-culture conditions for *Trichoderma atroviride* SG3403 and *B. subtilis* 22, resulting in improved antifungal metabolite production (Li et al. 2020).

The Central Composite Design (CCD), a robust and widely used RSM approach, was utilized for its ability to provide detailed model analysis with a limited number of experiments (Demirel and Kayan 2012). A total of 30 experimental runs were carried out to investigate the effects of four independent variables: pH, temperature, aeration, and agitation, on biomass and spore production. The model’s statistical significance and reliability were evaluated through analysis of variance (ANOVA), which identifies and quantifies the sources of variation in the dataset (El-Sayed et al. 2025). The models exhibited high statistical significance (*p* < 0.0001), confirmed by large Fisher’s *F*-values of 247.53 and 727.06 (*p*-value < 0.0001) biomass yield (g L⁻^1^) and spore yield (g⁻^1^), respectively, indicating that the variation explained by the model was much greater than the unexplained residual error (El-Sayed et al. 2024). The coefficients of determination (*R*^2^) were 0.98 for biomass yield and 0.997 for spore yield, indicating that 98% and 99.7% of the variability in biomass and spore yield responses, respectively, were accurately explained by the model. Values were all close to 1, demonstrating strong correlation and excellent agreement between observed and predicted responses (El-Sayed et al. 2020a; Kasiri et al. 2013). Furthermore, the slight difference between the adjusted and predicted *R*^2^ values (< 0.2) validates the model’s robustness and predictive precision. The Adequate Precision values of 54.0498 and 106.0863 for biomass and spore yield, respectively, all values exceeding the threshold of 4, indicated a high signal-to-noise ratio, whereas the low coefficients of variation (CV = 4.63% and 2.49%) for biomass yield and spore yield, respectively, reflected high reproducibility, good reliability, and precision of the experimental data (El-Sayed et al. 2020a).

ANOVA further revealed that pH (A), temperature (B), aeration(C), and agitation (D) were significant factors influencing biomass yield and spore yield (*p* < 0.05). These findings were visualized through three-dimensional (3D) surface plots generated by Design-Expert®, which depicted the interaction effects among variables and identified optimal conditions for maximum biomass and spore yield. In the contour maps, red-shaded zones represented regions of maximal biomass and spore yield, whereas blue zones corresponded to minimal production levels. The software’s numerical optimization tool identified the optimal combination of parameters, which were subsequently verified through experimental validation. The optimized conditions led to a 3.1-fold enhancement in biomass production and a 5.4-fold increase in spore yield, achieving a biomass concentration of 40.736 g L⁻^1^ and a spore density of 11.391 × 10^9^ g⁻^1^.

Diagnostic analyses further confirmed the robustness and adequacy of the statistical model. The normal probability plot of residuals (Fig. S3) indicated that the residuals were approximately normally distributed, supporting model validity. The Box–Cox transformation plot (Fig. S4) showed that no data transformation was required, as the current lambda (*λ* = 1) lay within the 95% confidence interval of the optimal *λ* value. Moreover, the predicted versus actual values plot (Fig. S5) revealed a strong agreement between experimental and predicted outcomes, while the residuals versus run plot (Fig. S6) displayed random scatter, confirming the absence of systematic errors or unmodeled variables affecting the response. Overall, the CCD-based RSM successfully optimized the fermentation parameters for *Lysinibacillus* sp. MK212927 resulting in a substantial improvement in biomass and spore yield production, and demonstrating the efficiency of this multivariate approach in microbial bioprocess optimization. In the present study, supplementation with *Lysinibacillus* sp. MK212927 was associated with a statistically significant increase in body weight compared with the infected and untreated groups, suggesting a potential beneficial physiological effect of the strain *in vivo*. However, it is important to note that body weight gain alone represents a phenotypic outcome and does not allow definitive conclusions regarding the underlying biological mechanisms. The absence of complementary measurements, including feed intake, feed conversion ratio, gut microbiota composition, inflammatory or metabolic biomarkers, and intestinal histopathology, represents a limitation of the present study. These parameters are critical for distinguishing whether weight gain arises from enhanced nutrient utilization, modulation of host–microbe interactions, attenuation of inflammation, or other metabolic effects. Therefore, while the observed trend is consistent with reports describing growth-associated benefits of *Bacillus*-related probiotics, such associations in the current study remain speculative and should not be interpreted as evidence of a causal mechanism (Marinova et al. 2019). In contrast, the reduction in body weight observed in rats infected with *E. coli* O157:H7 is consistent with previous reports linking enteric infection to intestinal inflammation, impaired nutrient absorption, and systemic metabolic stress. The transient weight changes observed in the antibiotic-treated group may similarly reflect temporary disruption of intestinal homeostasis rather than a sustained physiological effect. Taken together, these findings highlight the need for future studies incorporating detailed nutritional intake measurements, gut microbiota profiling, inflammatory and metabolic marker analysis, and histopathological examination of intestinal tissues to elucidate the mechanistic basis underlying the observed changes in body weight. Such investigations will be essential to confirm whether *Lysinibacillus* supplementation exerts growth-associated effects through modulation of intestinal health, host metabolism, or immune responses. Notably, *Lysinibacillus* outperformed *L. acidophilus*, possibly due to its spore-forming resilience and secretion of bioactive metabolites that enhance intestinal health and pathogen resistance (Ayyat et al. 2023). These findings collectively suggest that *Lysinibacillus* can promote host growth performance while mitigating infection-related weight loss, supporting its potential use as a functional probiotic strain. Collectively, these findings highlight the exceptional biosynthetic potential of *Lysinibacillus*, positioning it as a promising candidate for biotechnological exploitation in pharmaceutical, food, and medical applications.

## Conclusion

Comprehensive phenotypic assessments revealed that *Lysinibacillus* sp. MK212927 possesses a robust preclinical safety profile, confirming its non-pathogenic and non-toxic nature. Moreover, this study underscores the significant impact of strain-specific attributes on fermentation performance and outlines an optimized protocol for industrial-scale biomass and spore production. Collectively, these findings highlight the strong potential of *Lysinibacillus* as a promising probiotic candidate for human application, particularly in the development of dietary supplements, nutraceuticals, and therapeutic formulations.

## Supplementary Information

Below is the link to the electronic supplementary material.

ESM 1 (PDF 733 KB)

## Data Availability

The authors declare that the data supporting the findings of this study are available within the article and its supplementary information file. The 16S ribosomal RNA of **Lysinibacillus** isolate MK212927 was deposited into the NCBI GenBank under the accession number MK212927 ([https://www.ncbi.nlm.nih.gov/nuccore/MK212927](https://www.ncbi.nlm.nih.gov/nuccore/MT332429)) (accessed on 24 June 2024).
