# Supplementary material for: Probiotic characterization and spore production optimization of Lysinibacillus sp. MK212927
Source: Appl Microbiol Biotechnol. 2026 Mar 8;110(1):95. doi: 10.1007/s00253-026-13755-8 (PMC12971817; doi:10.1007/s00253-026-13755-8)
Supplement: Supplementary file 1 — (PDF 733 KB) [file 253_2026_13755_MOESM1_ESM.pdf]

Probiotic Characterization and Spore Production Optimization of *Lysinibacillus* sp. MK212927

Sayed E. El-Sayed[1]*, Albeir A. Messiha[2]

[1]Department of Microbiology and Immunology, Faculty of Pharmacy, Ahram Canadian University, POB: 12451, Sixth of October City, Giza, Egypt.

[2]Department of Inpatient Pharmacy, Virginia Hospital Center, Arlington, VA 22205, USA.

*** Corresponding Author: Sayed E. El-Sayed[1]**

Address: Department of Microbiology and Immunology, Faculty of Pharmacy, Ahram

Canadian University, POB: 12451, Sixth of October City, Giza, Egypt.

E-mail: sayed.emad@acu.edu.eg

Mobile: +2011-2637-5274

Authors' e-mails

Sayed E. El-Sayed: sayed.emad@acu.edu.eg

Albeir A. Messiha: messiha@vhchealth.org

Supplementary Methods

S1. Antipathogenic activity by agar well diffusion assay

The antipathogenic activity of *Lysinibacillus* isolate MK212927 was evaluated using an agar well diffusion assay adapted from Zhao et al. (2024).Eight human enteropathogenic bacterial strains (*Staphylococcus aureus* ATCC 29213, *Escherichia coli* O157:H7, *Bacillus cereus* ATCC 9634, *Bacillus subtilis* ATCC 6633, *Salmonella enteritidis* ATCC 13076, *Salmonella typhimurium* ATCC 14028, *Campylobacter jejuni* ATCC 29428, and *Clostridium perfringens* ATCC 13124) were revived from frozen stocks and cultivated in trypticase soy broth (TSB; HiMedia, Mumbai, India). Cultures were incubated for 24 h under optimal growth conditions specific to each organism. Following incubation, bacterial cultures were adjusted to an optical density of 0.1 at 600 nm (OD_{600}) using fresh growth medium to standardize the inoculum. A volume of 100 µL of each standardized bacterial suspension was evenly spread onto the surface of nutrient agar plates. Circular wells (10 mm in diameter) were aseptically punched into the agar using a sterile cork borer. Each well was loaded with 50 µL of an actively growing culture of *Lysinibacillus* isolate MK212927. Plates were incubated for 24 h under growth conditions appropriate for each test organism. After incubation, plates were examined for the presence of clear zones surrounding the wells, indicating inhibition of bacterial growth. The diameters of inhibition zones were measured in millimeters using a digital caliper, and results were recorded as the mean inhibition zone diameter. *Lactobacillus acidophilus* ATCC 43121 was included as a reference probiotic strain and tested under the same experimental conditions (Zhao et al. 2024).

S2. Sensitivity of the bacterium to antibiotics

The antibiotic susceptibility of *Lysinibacillus* sp. MK212927 was evaluated using the standard antibiotic disc diffusion method. An 18-hour broth culture of *Lysinibacillus* sp. MK212927 was prepared and adjusted to a turbidity equivalent to the 0.5 McFarland standard. A volume of 100 µL

of the standardized bacterial suspension was uniformly spread onto the surface of trypticase soy agar (TSA) plates (HiMedia, Mumbai, India). Antibiotic-impregnated discs (HiMedia, Mumbai, India) were aseptically placed onto the inoculated agar surface. The antibiotics tested included vancomycin (30 µg), azithromycin (15 µg), levofloxacin (5 µg), sulfamethoxazole (25 µg), doxycycline (30 µg), streptomycin (25 µg), and amoxicillin (25 µg). Following incubation under appropriate conditions, inhibition zone diameters were measured in millimeters. The susceptibility of the strain to each antibiotic was interpreted according to Clinical and Laboratory Standards Institute (CLSI) guidelines (2020, 2023) and classified as susceptible, intermediate (moderately susceptible), or resistant (Purkait et al. 2020).

S3. Tolerance to simulated gastrointestinal fluids and thermal stress

The tolerance of *Lysinibacillus* sp. MK212927 to gastrointestinal and thermal stress conditions was evaluated following previously described methods with minor modifications. Simulated gastric fluid (SGF) was prepared by dissolving porcine pepsin (2000 U/mL) in 0.2 M NaCl, and the pH was adjusted to 2.0 using hydrochloric acid. Moreover, simulated intestinal fluid (SIF) consisted of phosphate-buffered saline (PBS, pH 7.4) supplemented with ox-bile salts (0.3% w/v) and pancreatin (0.1% w/v). An aliquot of 100 µL of actively growing *Lysinibacillus* culture was added to 4.9 mL of SGF or SIF and incubated at 37 °C for 2 h with shaking at 200 rpm. Cell viability was determined by enumeration of colony-forming units (CFUs) before and after exposure to SGF or SIF. Survival was assessed based on changes in viable counts following treatment. In addition, actively growing bacterial cultures were subjected to heat treatment in a temperature-controlled water bath at 40 °C, 60 °C, 80 °C, and 100 °C for 30 min. Cell viability was evaluated by determining colony-forming units (CFUs) before and after thermal exposure. Heat tolerance was assessed based on survival following exposure to each temperature condition (Mazzantini et al. 2022).

S4. Antioxidant activity (DPPH scavenging assay)

Antioxidant activity of *Lysinibacillus* sp. MK212927 was determined using the DPPH (2,2-diphenyl-1-picrylhydrazyl) radical scavenging assay following Luang-In and Deeseenthum (2016). Briefly, 0.5 mL of *Lysinibacillus* culture was mixed with 3 mL of 0.05 mM DPPH solution prepared in absolute ethyl alcohol, while reference samples were prepared by replacing the bacterial culture with an equal volume of sterile broth mixed with DPPH solution. All assays were performed in triplicate and incubated in the dark at room temperature for 30 min to prevent light-induced DPPH degradation. Following incubation, samples were centrifuged at 8000 × g for 10 min to remove cell debris, and the absorbance of the supernatant was measured at 517 nm using a UV–visible spectrophotometer. L-ascorbic acid was used as a positive control, and *Lactobacillus acidophilus* ATCC 43121 was included as a reference probiotic strain for comparative assessment. Antioxidant activity was expressed as percentage DPPH scavenging activity (AA%) using the equation: **AA% = [1 − (A_sample / A_control)] × 100**, where *A_sample* represents the absorbance of the test reaction after blank correction and *A_control* represents the absorbance of the control reaction after correction (Luang-In and Deeseenthum 2016).

S5. Cell cytotoxicity (CCK-8 Assay)

The cytotoxic potential of *Lysinibacillus* sp. MK212927 was assessed in Caco-2 cells using the Cell Counting Kit-8 (CCK-8) assay (CytoTox 96 Non-Radioactive Cytotoxicity Kit, Promega, USA), following Zhang et al. (2024). Cell-free filtrates of *Lysinibacillus* sp. MK212927 and *Lactobacillus acidophilus* ATCC 43121 (reference probiotic control) were prepared by filtering 24-h fermented cultures through 0.22 µm syringe filters. Caco-2 cells were exposed to the filtrates and incubated at 37 °C, after which 10 µL of CCK-8 reagent was added to each well and plates were incubated for an additional 4 h. Background absorbance was corrected using wells containing complete Dulbecco's Modified Eagle Medium (DMEM) alone or medium supplemented with filtrates but without cells to account for color interference. Untreated Caco-2 cells incubated with

phosphate-buffered saline served as the negative control, while cells cultured in complete DMEM served as the positive control. Absorbance was measured at 450 nm, and cell viability was expressed as the percentage of viable cells relative to the untreated control (Zhang et al. 2024).

S6. Adhesion Capacity Assessment

The adhesion capacity of *Lysinibacillus* sp. MK212927 was evaluated using an in vitro adhesion assay with human Caco-2 cells following Sim et al. (2024). Caco-2 cells were seeded at a density of 2.8×10^4 cells/cm^2 in 12-well tissue culture plates and maintained in Dulbecco's Modified Eagle Medium (DMEM) supplemented with 10% heat-inactivated fetal bovine serum, non-essential amino acids, and L-glutamine, with daily medium renewal. Cells were allowed to differentiate for 21 days to reach the late post-confluent stage, and antibiotic-free DMEM was used during the final 24 h before the assay. *Lysinibacillus* cultures were grown overnight, harvested by centrifugation, washed twice with sterile phosphate-buffered saline (PBS), and resuspended in antibiotic-free DMEM to a standardized concentration of 1×10^8 CFU/mL. Differentiated Caco-2 monolayers were inoculated with 1 mL of the bacterial suspension and incubated for 2 h at 37 °C in a humidified atmosphere containing 5% CO_2. Non-adherent bacteria were removed by washing the monolayers three times with sterile PBS, after which adherent bacteria were recovered by lysing epithelial cells with 0.1% (v/v) Triton X-100 in PBS for 10 min at room temperature. The lysates were serially diluted, and viable bacteria were enumerated using the drop-plate method on appropriate agar media. Adhesion capacity was calculated as the percentage of adhered bacteria relative to the initial inoculum $(CFUrecovered/CFUinitiallyadded) \times 100$. All experiments were performed in triplicate, and results were expressed as mean ± standard deviation (Sim et al. 2024).

S7. Assessment of Biomass and Spore Production

One mL of each culture was transferred to 9 mL of sterile normal saline, vortexed, and heated at 80 °C for 30 minutes to eliminate vegetative cells. The treated samples were serially diluted tenfold, and 100 µL aliquots from the 10^6–10^8 dilutions were spread on sterile agar plates. Plates were incubated for 24–48 hours, after which colony counts were performed to estimate the spore concentration (spores mL^{-1}). Standard Gram and spore staining techniques were used to verify sample purity and spore quality (Khardziani et al. 2017).

Table S1. Central composite design runs for the 4 different factors tested, showing the observed responses

Run	A: pH	B: Temperature °C	C: Agitation rate (rpm)	D: Aeration rate (vvm)	Biomass yield (g L^{-1})	Spores yield ×10^9 $(g)^{-1}$
1	6.5	37	150	0.5	45.2	8.08
2	6.5	37	150	0.5	45.8	8.08
3	5	30	100	0.8	12.1	12.26
4	5	44	100	0.2	18.5	4.8
5	5	44	200	0.8	35.6	6.2
6	6.5	44	150	0.5	38.9	4.51
7	6.5	37	150	0.5	45.5	8.08
8	5	30	200	0.2	22.3	15.5
9	6.5	37	150	0.2	41.7	8.54
10	5	37	150	0.5	32.8	9.69
11	8	30	100	0.2	15.2	9.6
12	8	44	100	0.8	28.4	3.86
13	6.5	37	150	0.8	43.1	7.61
14	5	44	200	0.2	31.2	5.07
15	8	44	200	0.2	34.8	3.19
16	8	44	100	0.2	24.6	2.61
17	6.5	37	200	0.5	47.3	8.25
18	5	44	100	0.8	26.9	6.1

19	8	30	200	0.2	25.7	10
20	8	30	200	0.8	29.5	8.11
21	8	44	200	0.8	42.6	4.26
22	6.5	37	150	0.5	45	8.08
23	6.5	30	150	0.5	28.4	11
24	8	37	150	0.5	36.2	6.46
25	6.5	37	100	0.5	38.1	7.9
26	6.5	37	150	0.5	45.6	8.08
27	6.5	37	150	0.5	44.9	8.08
28	5	30	100	0.2	10.8	15.21
29	5	30	200	0.8	26.3	12.38
30	8	30	100	0.8	20.1	7.69

Table S2. Effect of probiotic and antibiotic treatments on rat body weight (g) over three weeks

**Test Group**	**Initial Weight (g)**	**1-Week Feeding (g)**	**2-Week Feeding (g)**	**3-Week Feeding (g)**
Control	220 ± 7.21	236.0 ± 7.42	239.0 ± 11.51	243.2 ± 12.49
Antibiotic	220 ± 7.21	230.0 ± 13.42	236.0 ± 23.02	239.8 ± 37.75
Lysinibacillus sp. MK212927	220 ± 6.52	242.6 ± 9.73	269.0 ± 11.32	355.3 ± 21.72
E. coli O157:H7	220 ± 3.65	213.0 ± 7.58	200.6 ± 19.17	189.9 ± 34.85
L. acidophilus ATCC 43121	220 ± 3.65	230.2 ± 10.73	259.0 ± 16.34	334.6 ± 19.54

Values are presented as mean ± SD (n = 5).

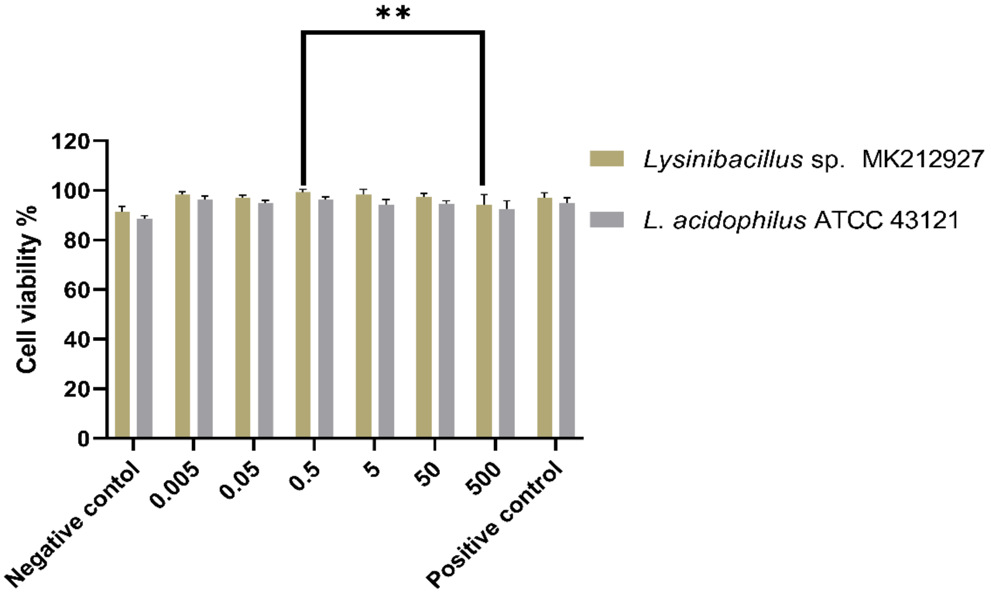

Figure S1. Effects of *Lysinibacillus* sp. MK212927 and *L. acidophilus* ATCC 43121 cell-free filtrates on the viability of Caco-2 cells

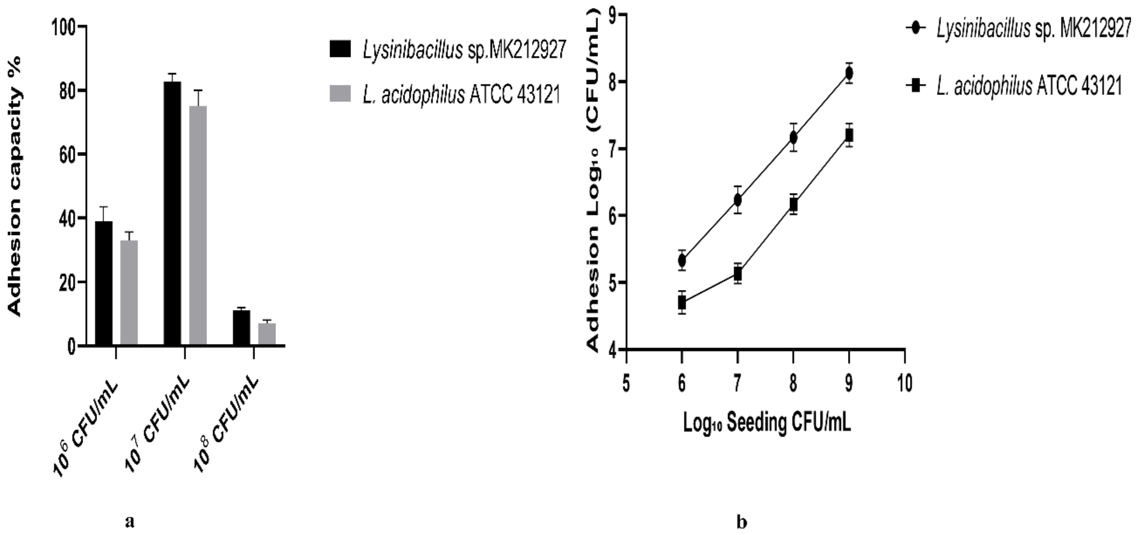

Figure S2. Adhesion capacity of *Lysinibacillus* sp. MK212927 and *L. acidophilus* ATCC 43121 to Caco-2 cells at different initial seeding concentrations

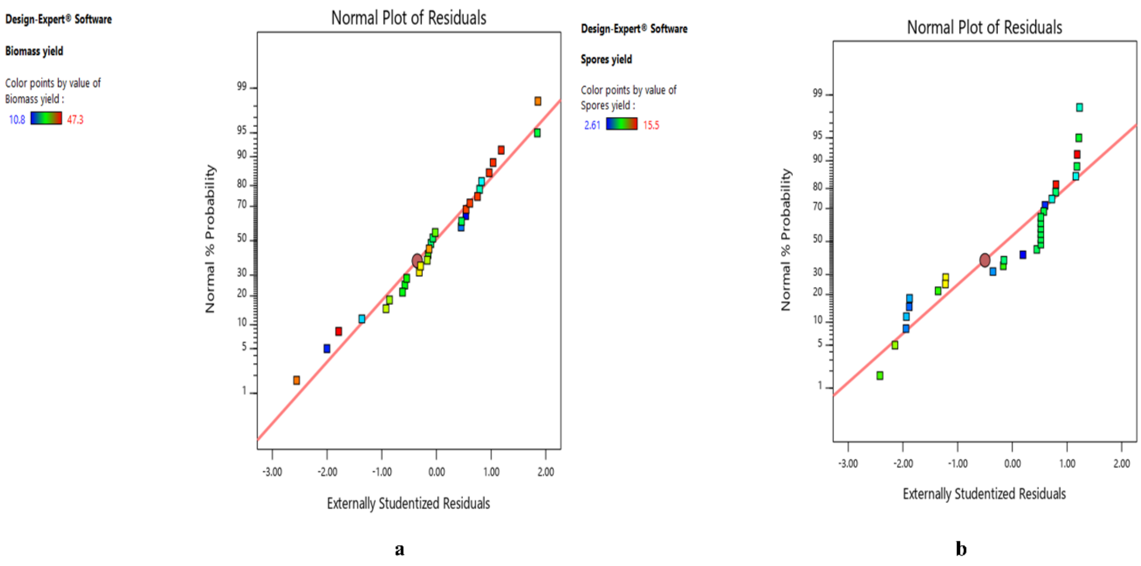

Figure S3. The normal probability plots of residuals for (a) biomass, and (b) spores yield.

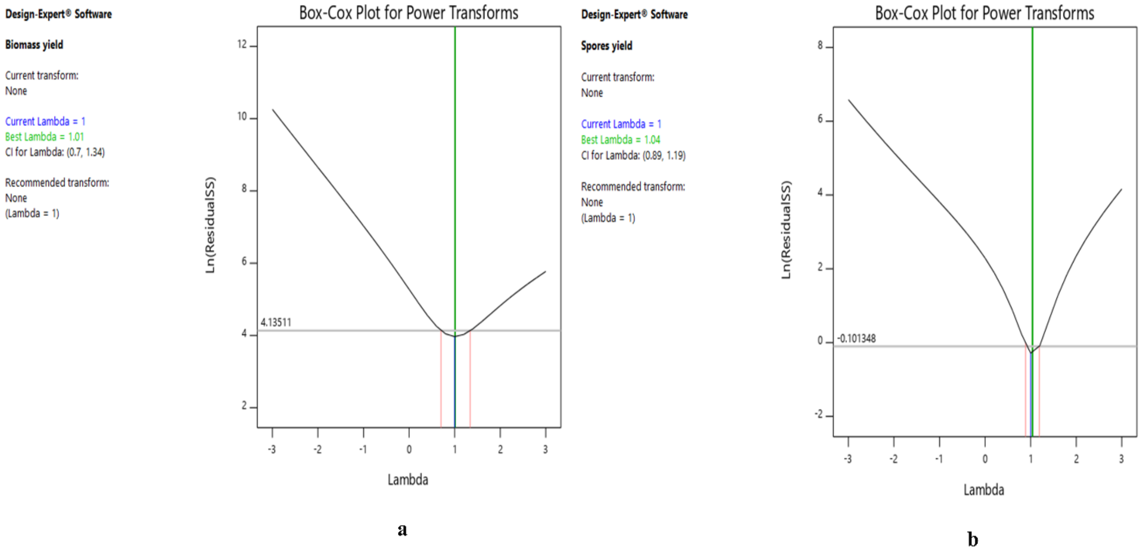

Figure S4. The Box-Cox plots for (a) biomass, and (b) spores yield.

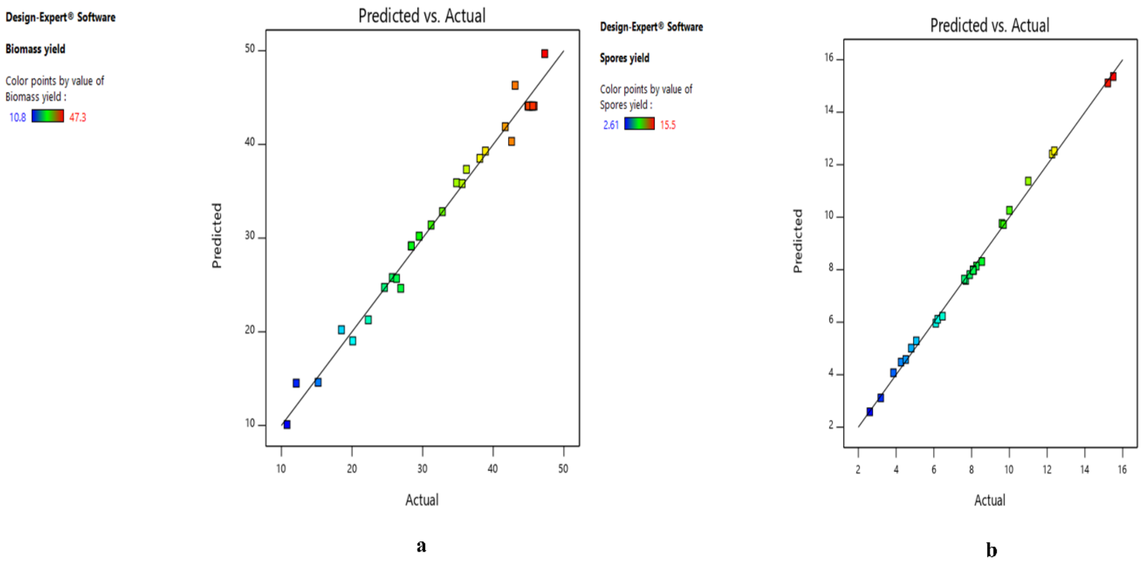

Figure S5. The predicted versus actual values plots for (a) biomass, and (b) spores yield.

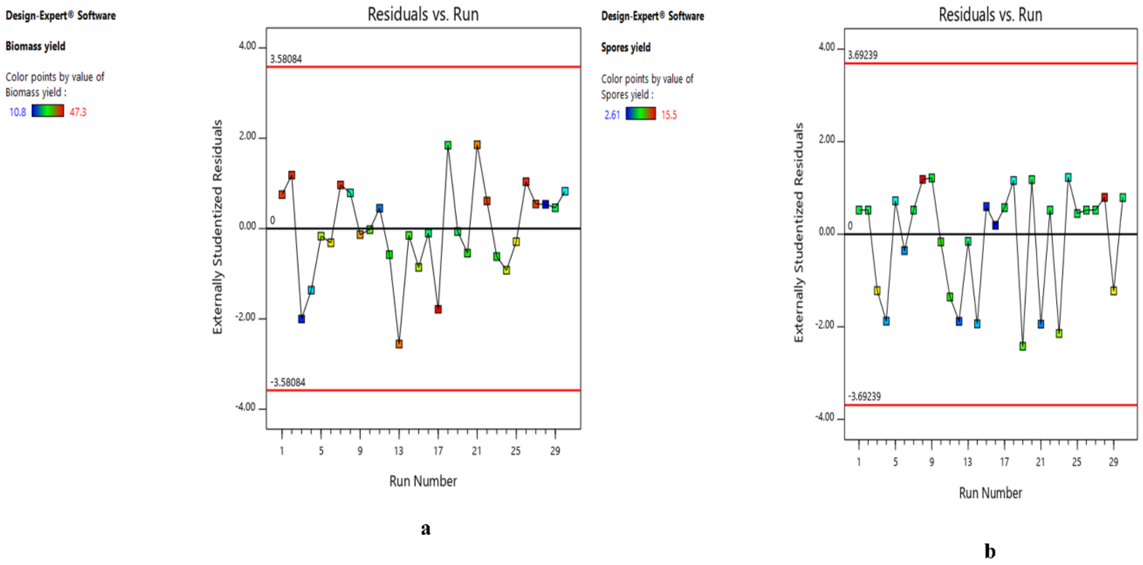

Figure S6. The residuals versus run number plots for (a) biomass, and (b) spores yield.

References

Khardziani T, Sokhadze K, Kachlishvili E, Chistyakov V, Elisashvili V (2017) Optimization of enhanced probiotic spores production in submerged cultivation of *Bacillus amyloliquefaciens* B-1895. J Microbiol Biotechnol Food Sci 7(2):132–136.

Luang-In V, Deeseenthum S (2016) Exopolysaccharide-producing isolates from Thai milk kefir and their antioxidant activities. LWT 73:592–601. https://doi.org/10.1016/j.lwt.2016.06.086

Mazzantini D, Calvigioni M, Celandroni F, Lupetti A, Ghelardi E (2022) In vitro assessment of probiotic attributes for strains contained in commercial formulations. Sci Rep 12(1):21640. https://doi.org/10.1038/s41598-022-25688-z

Purkait S, Bhattacharya A, Bag A, Chattopadhyay RR (2020) Synergistic antibacterial, antifungal and antioxidant efficacy of cinnamon and clove essential oils in combination. Arch Microbiol 202(6):1439–1448. https://doi.org/10.1007/s00203-020-01858-3

Sim EA, Kim SY, Kim S, Mun EG (2024) Probiotic potential and enhanced adhesion of fermented foods-isolated lactic acid bacteria to intestinal epithelial Caco-2 and HT-29 cells. Microorganisms 13(1):32. https://doi.org/10.3390/microorganisms13010032

Zhang Y, Cao T, Wang Y, Yang R, Han Y, Li S, Liu D, Yue Y, Cao Y, Li B, Wang S, Huo G (2024) Effects of viable and heat-inactivated *Bifidobacterium longum* D42 on proliferation and apoptosis of HT-29 human colon cancer cells. Foods 13(6):958. https://doi.org/10.3390/foods13060958

Zhao Z, Li W, Tran TT, Loo SCJ (2024) *Bacillus subtilis* SOM8 isolated from sesame oil meal for potential probiotic application in inhibiting human enteropathogens. BMC Microbiol 24(1):104. https://doi.org/10.1186/s12866-024-03263-y
